# Efficacy and safety of subcutaneous immunotherapy in asthmatic children allergic to house dust mite: a meta-analysis and systematic review

**DOI:** 10.3389/fped.2023.1137478

**Published:** 2023-06-15

**Authors:** Chen Zheng, Hao Xu, Shumin Huang, Zhimin Chen

**Affiliations:** Department of Pulmonology, The Children's Hospital, Zhejiang University School of Medicine, Hangzhou, China

**Keywords:** subcutaneous immunotherapy, asthma, children, house dust mite, efficacy, safety, systematic review, meta-analysis

## Abstract

**Background:**

Subcutaneous immunotherapy (SCIT) has been proved to be effective and safe in adult asthma. But it is still controversial in children.

**Object:**

To evaluate the efficacy and safety of SCIT in asthmatic children with allergy to house dust mite.

**Method:**

We searched the databases of Cochrane Library, EMBASE and MEDLINE (from 1 January 1990 to 1 December 2022). Two reviewers independently screened studies, extracted data and critically appraised the risk of bias. We used the Revman 5 to synthesize the effect sizes.

**Results:**

We finally selected 38 eligible studies including 21 randomized controlled trials to evaluate the efficacy and safety of SCIT and 17 observational studies to assess the safety. The results revealed that short-term asthma symptom scores were declined with a standardized mean difference (SMD) of −1.19 (95% CI: −1.87, −0.50) in 12 researches with high heterogeneity. Short-term asthma medication scores were decreased with SMD −1.04 (95% CI: −1.54, −0.54) in 12 heterogeneous researches. One study showed no significant reduction in combined symptom and medication scores without providing details. No studies we reviewed reported long-term efficacy. SCIT resulted in an obviously increased risk of adverse reactions compared with placebo. For secondary outcomes, SCIT improved life quality and reduced the numbers of annual asthma attacks and allergen-specific airway hyperreactivity, but without significant improvement in pulmonary function, asthma control or hospitalization.

**Conclusions:**

SCIT can reduce the short-term symptom scores and medication scores regardless of different treatment duration or mono/polysensitization, but with an increased incidence of local and systemic adverse effects. Further studies on pediatric asthma are needed to evaluate the long-term efficacy, and clarify the effectiveness of SCIT in specific population using mix allergen extracts or with severe asthma. Overall, it is recommended for children with mild-moderate HDM-driven allergic asthma.

## 1. Introduction

Allergic asthma (AA) has been one of the most common chronic diseases among children with an uprising prevalence in recent years. Most patients can benefit from avoidance strategies, drug treatment and allergen immunotherapy (AIT) (1). However, house dust mite (HDM), one of the most relevant triggers of allergic diseases worldwide, is difficult to be avoided (2). Thus, it is necessary to treat allergic disease due to HDM by using the other two approaches. AIT is a highly attractive therapy method to AA by its disease-modifying effect which can exist for a long time after discontinuation (1, 3–5). The main administrations of AIT are subcutaneous immunotherapy (SCIT) and sublingual immunotherapy (SLIT). Limited trials compared the outcomes of SCIT with SLIT head-to-head, offering low-grade evidence to support greater benefits of SCIT (6–9). In indirect comparison to SLIT, SCIT appeared to be more effective in controlling asthma symptoms and decreasing medication use (10). However, regarding the low adherence, individual variation and safety, the evidence supporting SCIT in the pediatric population is still insufficient and controversial (1, 11, 12), making it remain underused in children (13).

In order to solve these problems, several high-quality trials have provided evidence on the efficacy of AIT in asthmatic children (14–16). However, it is still difficult to draw robust conclusions due to the disparities in outcome definitions and the heterogeneity of interventions when analyzing pooled data (9). Considering the limitations of meta-analyses, the way of focusing on studies of single-product or sub-population may hold promise (1). So, it might be possible to come to more convincing or homogeneous conclusions by focusing exclusively on HDM extracts for pediatrics with mite allergy, while no meta-analyses has reported such relevant results to date. Therefore, we conducted the research to pool data in regard to the asthmatic children allergic to HDM, which may be helpful to promote the rational use of SCIT in clinical practice.

## 2. Method

According to the guidelines for systematic reviews, two reviewers screened studies, extracted data and critically appraised the risk of bias all alone. And when necessary, they would consult a third reviewer. A detailed description of the methods has previously been posted online (17).

### 2.1. Data searches and study selection

We searched the databases of Cochrane Library (both Cochrane Systematic Reviews and The Cochrane Central Register of Controlled Trials), MEDLINE and EMBASE (from 1 January 1990 to 1 December 2022) according to the principle of PICOS. All references we searched were uploaded to the reference management software named NoteExpress (v3.5.0.9054, Aegean Software, Beijing, China) for initial deduplication and management. Two reviewers independently screened eligible studies that meet the following inclusion criteria:

**Population:** children under 18 years of age with allergic asthma (diagnosed by physicians according to accepted diagnostic criteria) and evidence of clinically relevant allergic sensitization to house dust mites as determined by an objective biomarker (e.g., skin prick test or specific-IgE).
**Intervention:** any way of SCIT (e.g., conventional SCIT, rush SCIT or cluster SCIT) using specific allergen extracts of house dust mite with/without other allergens.
**Comparator:** placebo or conventional drug therapy when comparing the effectiveness of SCIT. There could be no comparison group when comparing the safety of SCIT in descriptive studies.
**Outcomes:** short-term (during treatment) and long-term (at least one year after discontinuation of SCIT) efficacy assessed by the improvement of asthma symptoms and medication use as the primary outcomes as well as the safety reported by incidence of adverse effects; quality of life using the Asthma Quality of Life Questionnaire (AQLQ) or other appropriate tools, pulmonary function, asthma control showing the extent to which the various manifestations of asthma were reduced or removed by SCIT (18), and specific or nonspecific bronchial provocation test as secondary outcomes.
**Study Design:** Randomized controlled trials (RCTs) were included to assess the efficacy of SCIT in asthmatic children and these were supplemented with descriptive studies for the evaluation of adverse effects. Systematic reviews and meta-analysis were also selected for further screening.

If only adults or partially (<60% of totality) eligible participants meet all the inclusion criteria involved, such a study would be excluded. Besides, the language of original articles was limit in English and Chinese.

### 2.2. Data extraction and quality assessment

Two reviewers independently extracted information from the original papers by a previously designed data extraction sheet. During the step, risk of bias assessment was simultaneously processed on each randomized controlled trial (RCT) using the Cochrane Collaboration's tool (19). Those descriptive studies about the safety of SCIT were analyzed separately without quality assessment. The discrepancies were discussed together and settled by a third reviewer if disagreements remained.

### 2.3. Data synthesis

The outcomes we focused on were the efficacy and safety of SCIT in pediatric asthma. For the efficacy outcomes which were mainly continuous variables, we used the mean difference (MD), or SMD if appropriately, to represent the effect size with a 95% confidence interval (CI). And for the safety, as a dichotomous variable, relative risk (RR) of local and systemic adverse reactions were applied. We used Revman 5.0 (version 5.4.1) to synthesize these effect sizes. Heterogeneity is quantified using *I*^2^ and categorized as no importance (0% ≤ *I*^2 ^≤ 30%),mild heterogeneity (30% < *I*^2 ^< 50%), moderate heterogeneity (50% ≤ *I*^2 ^≤ 75%) and substantial heterogeneity (75% < *I*^2 ^≤ 100%). If the heterogeneity was significant (*I*^2 ^≥ 50%), the random effect model would be selected.

### 2.4. Analysis of subgroups

In light of the large heterogeneity of previous RCTs about the relevant topic, subgroups analyses seemed to be sensible and necessary to investigate and reduce the heterogeneity. Lots of previous studies have indicated that some population demographics such as monosensitization or polysensitization, severity of asthma, treatment duration, etc., can influence the effectiveness of SCIT (20). Therefore, subgroup analyses were undertaken to compare: monosensitization vs. polysensitization, mild or moderate vs. severe asthma, the duration of treatment, and single HDM allergen vs. mixed allergens. In accordance with EMA suggestion, we defined a single HDM allergen as one allergen or mixed homologous allergens of mites (e.g., *Dermatophagoides farinae*, *Dermatophagoides pteronyssinus* and *Blomia tropicalis*) and mixed allergens as a mixture of different species (e.g., Dermatophagoides species, grass pollen and *Alternaria alternata*).

## 3. Results

**Figure 1:**
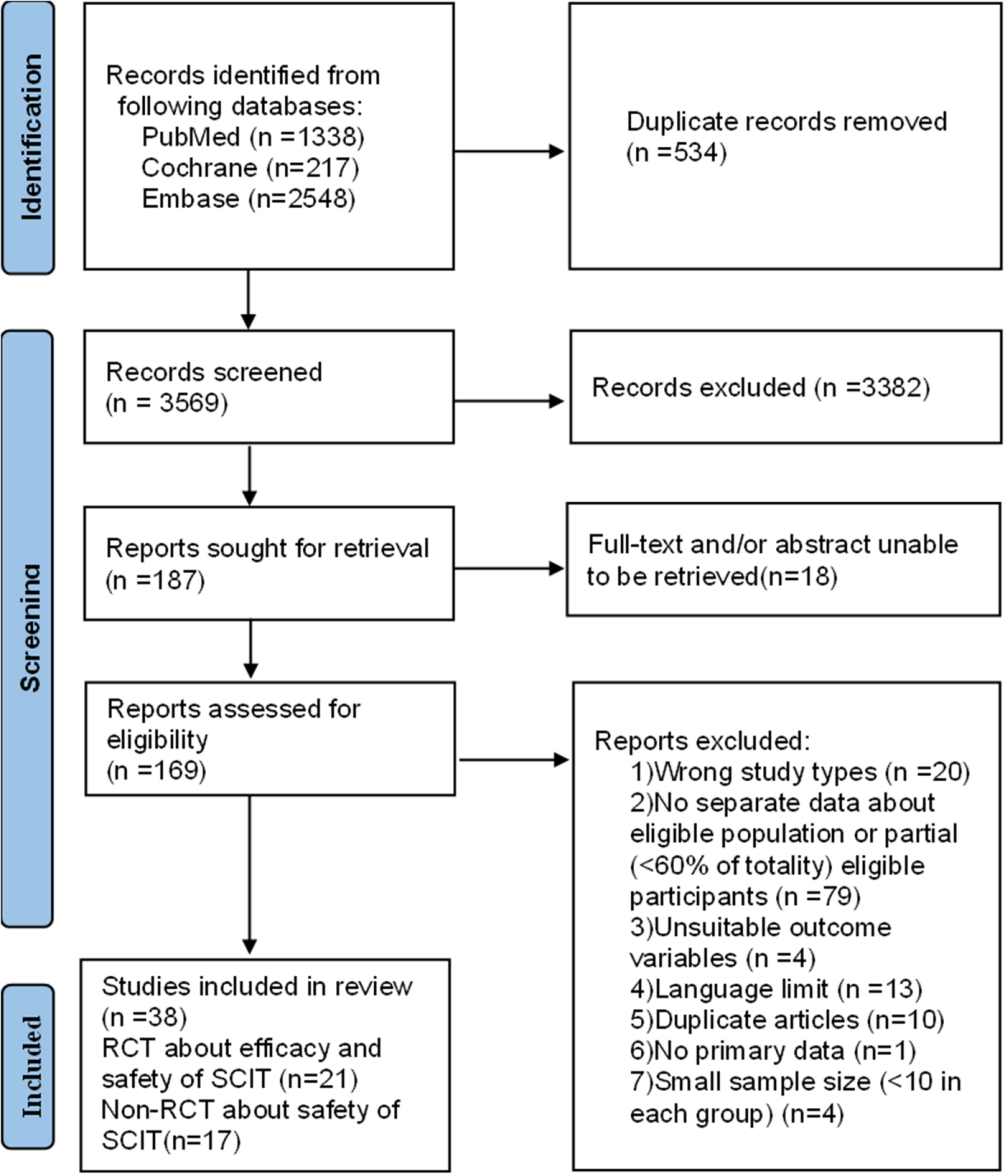
The flow chart of data searches and study selection.

After the identification and screening, we included 38 eligible researches (21 RCTs for efficacy or safety and 17 non-RCTs about the safety of SCIT) (7, 8, 14–16, 21–53) (Figure 1). Among these, five double-blind and placebo-controlled (DBPC) trials assessed both the efficacy and safety of the SCIT in HDM-sensitized asthmatic children. Each original article exclusively focused on children except two studies including both children and adults. Most included subjects suffered from mild to moderate asthma according to GINA guideline. In addition to asthma, some patients were concomitant with allergic rhinoconjunctivitis or eczema in the majority of included studies. Characteristics of included researches are summarized in Supplementary Tables S1,S2. The risk of bias assessment is shown in Supplementary Table S3.

### 3.1. Primary outcomes

Based on the literature reviewed and guidelines recommended, we included asthma symptom scores, asthma medication scores, and combined symptom and medication scores (CSMS) as the primary outcomes. A total of 14 studies evaluated the efficacy of SCIT using asthma symptom scores, but only 12 of them reported relevant data. In all reported studies, the four basic asthma symptoms (cough, wheezing, breathlessness and dyspnea) were assessed and recorded on a 4-point scale, except for three studies in which the maximum score was 5 or 20 points (24, 29, 30). In addition, visual analog scale was also recorded to evaluate the severity of asthma and rhinitis symptom in five studies, which revealed a significant improvement. However, we did not pool data from these studies because only two of them reported the variance in detail. For the definition of asthma medication scores, it varied in most included trials, depending on the type and dosage of medication, and the rating scale ranged from 2 to 10 points. As recommended in guidelines, CSMS is an appropriate outcome to evaluate clinical effectiveness and was reported in one study as the sum of asthma medication scores and asthma symptom scores. In order to compare the efficacy of SCIT in various trials, the mean change in these primary outcomes between baseline and the last follow-up visit was calculated as the effect variable.

#### 3.1.1. Short-term asthma symptoms scores

**Figure 2:**
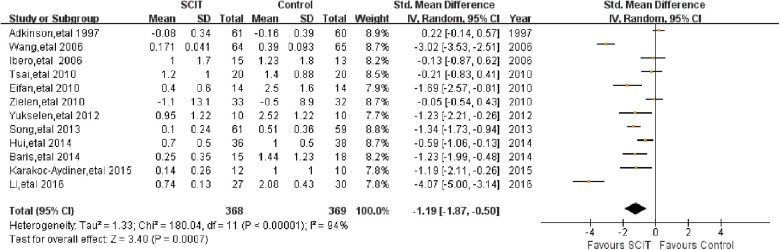
Meta-analysis of RCTs for short-term asthma symptom scores comparing SCIT and control groups (random-effects model).

Twelve trials reported on the short-term asthma symptom scores which were obtained during the SCIT treatment to evaluate the efficacy of SCIT. We pooled data from those studies and the SMD was −1.19 (95% CI: −1.87, −0.50; see Figure 2), indicating that SCIT could improve symptom scores significantly compared to placebo or medication therapy. The efficacy was confirmed after excluding studies at high risk of bias (ROB). However, substantial heterogeneity was obviously among studies (*I*^2^ = 94%).

##### 3.1.1.1. Subgroup analyses

**Figure 3:**
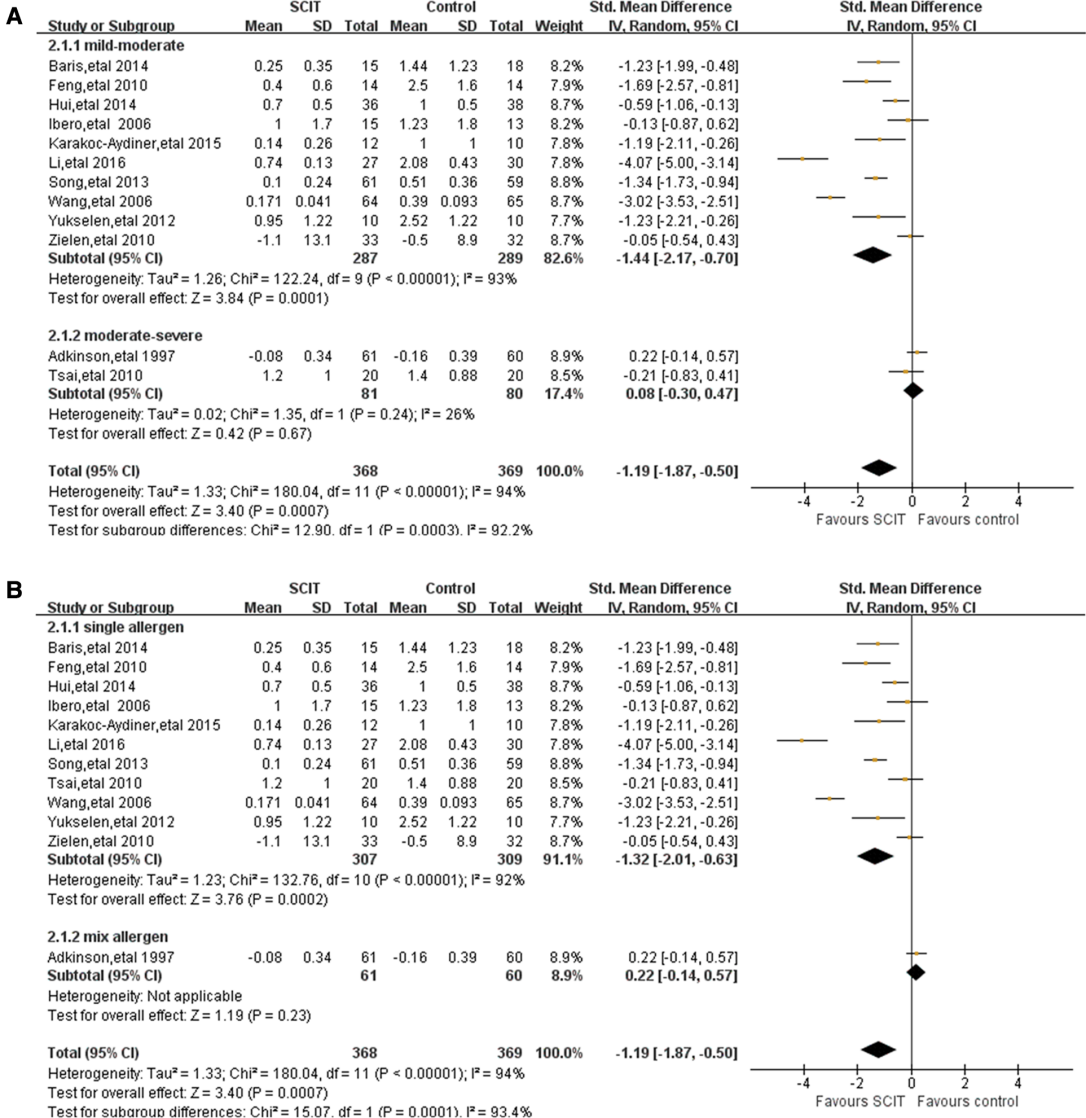
Subgroup analyses of short-term asthma symptom scores comparing SCIT and control groups: (**A**) mild-moderate asthma vs. moderate-severe asthma; (**B**) single allergen vs. mix allergens.

**Mild-moderate asthma vs. moderate-severe asthma:** this analysis suggested an apparent efficacy of SCIT on mild-moderate asthma with SMD −1.44 (95% CI: −2.17, −0.70) while SMD 0.08 (95% CI: −0.30, 0.47) on moderate-severe asthma (see Figure 3A). This indirect comparison indicated that mild cases could benefit more compared with severe asthmatic children. However, the result was still doubtful because of the large heterogeneity and the small quantity of studies in subgroups.
**Single allergen vs. mixed allergens:** the evidence of benefit for SCIT was found obviously in patients using single allergen with SMD −1.32 (95% CI: −2.01, −0.63), but still lacking in those with mixed allergens SMD 0.22 (95% CI: −0.14, 0.57) (see Figure 3B). It seemed that single HDM SCIT could be more effective on the asthma symptom control than mixed allergens SCIT, which still needs further researches to support.
**Mono-sensitivity vs. poly-sensitivity& Treatment duration:** there is evidence of large benefit of HDM SCIT both in mono-sensitized patients and poly-sensitized population. Similar result was showed in patients under SCIT treatment for more than or less than 3 years, supporting the effectiveness of SCIT during different duration. However, high heterogeneity still existed after subgroup analyses.

#### 3.1.2. Short-term asthma medication scores

**Figure 4:**
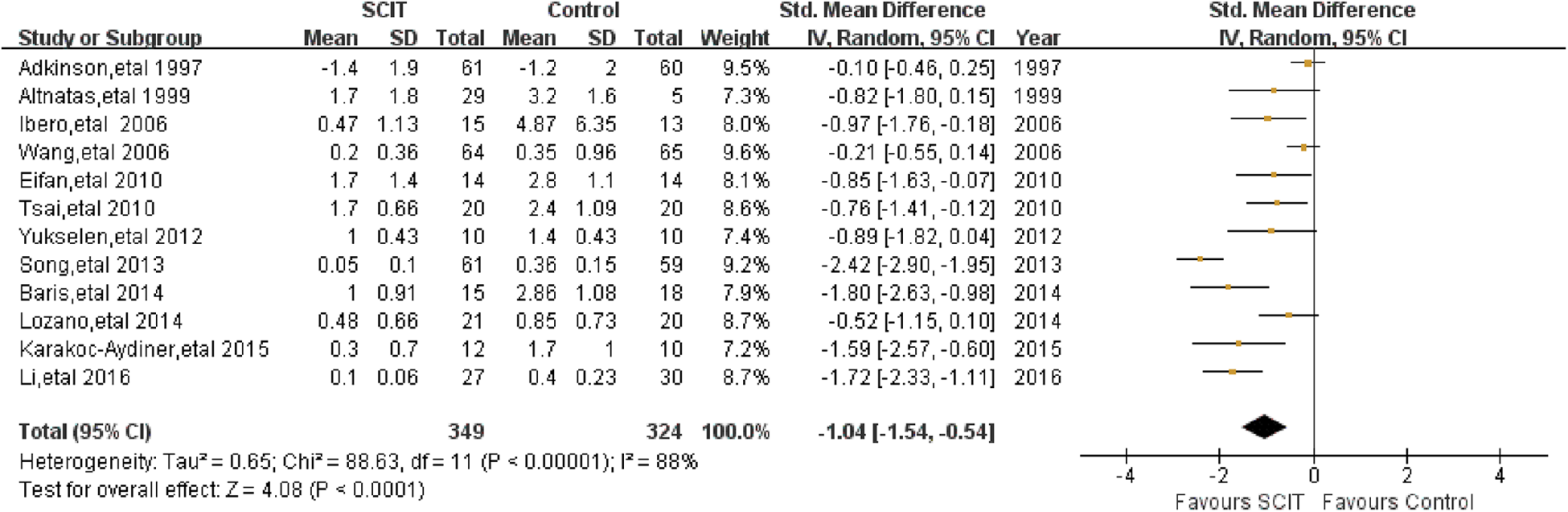
Meta-analysis of RCTs for short-term asthma medication scores comparing SCIT and control groups (random-effects model).

Twelve heterogeneous studies reported the asthma medication scores in short term. The pooled data demonstrated a statistically significant reduction in asthma drug usage with SMD −1.04 (95% CI: −1.54, −0.54) (Figure 4), which was confirmed again after the sensitivity analysis.

##### 3.1.2.1. Subgroup analyses

**Mild-moderate asthma vs. moderate-severe asthma:** the analysis revealed that SCIT is apparently efficacious in mild to moderate cases with SMD −1.18 (95% CI: −1.75, −0.61; *I*^2 ^= 87%), but not in moderate to severe asthmatic cases with SMD −0.37 (95% CI: −1.01, 0.26; *I*^2 ^= 68%).
**Single allergen vs. mixed allergens:** there is evidence of SCIT beneficial to patients with single allergen SMD −1.14 (95% CI: −1.66, −0.62; *I*^2 ^= 85%) and a possible benefit in those with mixed allergens SMD −0.10 (95% CI: −0.46, 0.25). This result needs to be cautiously interpreted on account of limited studies in subgroups (only one study about mixed allergens).
**Mono-sensitivity vs. poly-sensitivity & Treatment duration:** both obvious efficacy of SCIT can be found in either sub-group. Those who received SCIT lasting more than 3 years could benefit more than the opposite.

#### 3.1.3. CSMS

Only one study showed no significant reduction in CSMS without providing data in details (21).

#### 3.1.4. Long-term symptom scores and medication scores

No relevant studies we reviewed reported these two outcomes which were evaluated at least one year after discontinuation of SCIT.

#### 3.1.5. Safety

**Figure 5:**
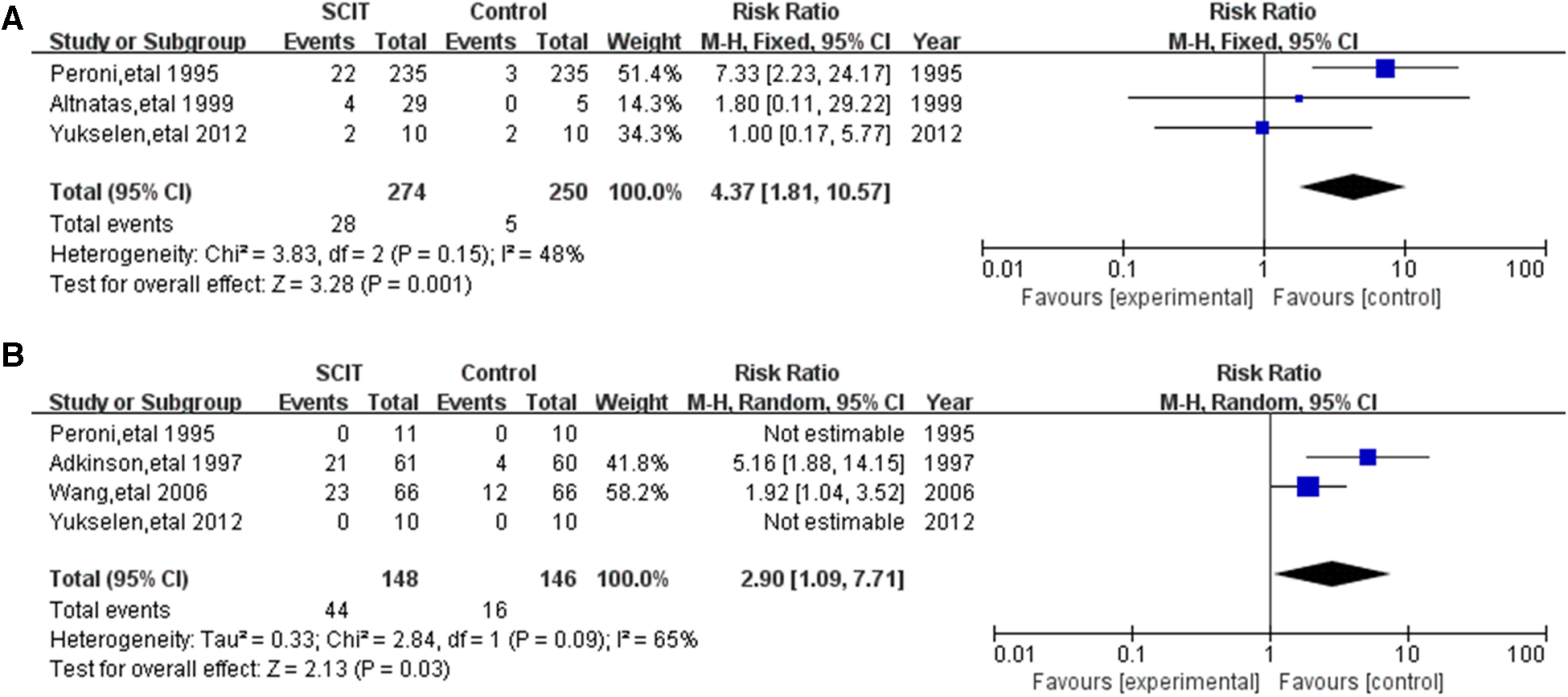
Meta-analysis of double-blind and placebo controlled trials for local and systemic adverse events comparing placebo groups: (**A**) local adverse events (fix-effects model); (**B**) systemic adverse events (random-effects model).

Five DBPC trials assessed adverse events (AEs) of the SCIT in HDM-sensitized asthmatic children. 3 mildly heterogeneous trials of those reported the local AEs and 4 moderately heterogeneous trials reported the systemic AEs. Compared to placebo, SCIT could increase the risk of local and systemic AE, with RR 4.37 (95% CI: 1.81, 10.57) and 2.90 (95% CI: 1.09, 7.71) respectfully (Figure 5). Subgroup analysis was impractical in those limited studies. In the total 33 researches for the safety, varied in participants characteristics or interventions, the incidence of local AE ranged from 1.3% to 64.8% of total injections, while systemic AE mostly accounted for less than 5% which were mainly mild or moderate without dead cases (see Supplementary Table S2). Most participants in those researches were elder than 5 years old and suffered from mild-moderate asthma. Single HDM allergen/allergoid with aluminum as adjuvant was the most common formulation. One study in Japan showed higher incidence of systemic AE up to 10.4% in patients under 5 years old, which might be exaggerated because it only reported the adverse events in induction phase (46). There are also some other studies evaluating the safety in children younger than 5 years old, which did not show the significant difference in contrast to elder children (21, 50).

### 3.2. Secondary outcomes

#### 3.2.1. Pulmonary function

##### 3.2.1.1. FEV1

Nine studies reported FEV1 with moderate heterogeneity. The meta-analysis demonstrated a borderline improvement of SCIT on the FEV1 compared with the placebo or pharmacotherapy by random effects model with MD 3.37 (95% CI: 0.23, 6.51; *I*^2 ^= 57%). However, the evidence is not robust after sensitivity analysis.

**Figure 6:**
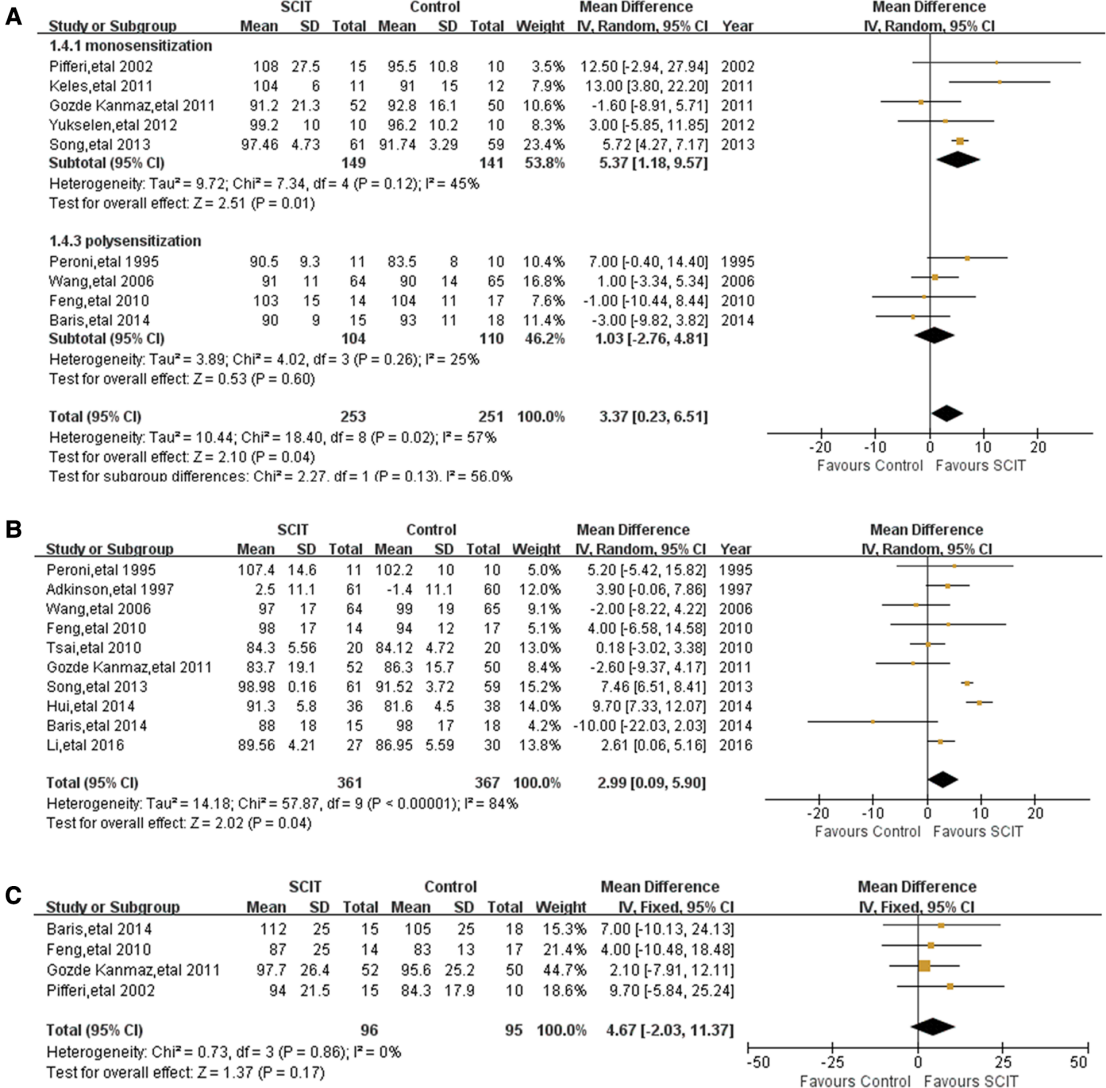
Meta-analysis of RCTs for pulmonary function comparing SCIT and control groups: (**A**) FEV1 after subgroup analysis of monosensitization vs. polysensitization (random-effects model); (**B**) PEF (random-effects model).; (**C**) MMEF (fix-effects model).

There is mildly heterogeneous evidence of large benefit of HDM SCIT only in mono-sensitized patients MD 5.37 (95% CI: 1.18, 9.57; *I*^2 ^= 45%, see Figure 6A). Ineffectiveness of SCIT was showed in polysensitized patients, either mild-moderate or moderate-severe asthmatic children and patients treated for different duration. All the participants included in those studies were treated with single allergen of HDM.

##### 3.2.1.2. PEF

Pooled data from 10 trials reporting on PEF demonstrated a marginal improvement of SCIT with a MD of 2.99 (95% CI: 0.09, 5.90; *I*^2 ^= 84%; see Figure 6B). However, the sensitivity analysis showed the lack of robustness of the finding again. There is no clear evidence supporting that SCIT could apparently improve the value of PEF in a certain population except those treated more than 3 years after subgroup analyses.

##### 3.2.1.3. MMEF

Four homogeneous studies reported the value of MMEF, from which the pooled data showed a suggested efficacy (but not confirmed) of SCIT MD 4.67 (95% CI: −2.03, 11.37; see Figure 6C). The evidence was still convincing after sensitivity analysis. Subgroup analyses were not performed because of few studies related.

#### 3.2.2. Quality of life

There were two studies unable to be pooled reporting the Asthma Quality of Life Questionnaire (AQLQ), which revealed an apparent improvement compared with the control group. Another study evaluated the outcome using Pediatric Asthma Caregiver Quality of Life Questionnaire (PACQOL), which also illustrated the efficacy of SCIT in improving asthma related quality of life (21).

#### 3.2.3. Asthma control

Two studies reported the outcome on asthma symptoms control using numerical or categorical tools. Only one article performed the asthma control test (ACT) to assess the asthma control (22). The other research used 11-items of asthma control parameters according to GOAL criteria (54). Neither of them found significant difference between the SCIT and control group regarding asthma control.

#### 3.2.4. Exacerbation

Eight studies reported information about asthma exacerbation in different definitions. Three studies, of substantial heterogeneity, reported on exacerbation which was defined by the number of annual asthma attacks. Pooling of data from those studies showed significant difference between the SCIT and control therapy with SMD −1.07 (95% CI: −1.92, −0.22; *I*^2 ^= 80%), which showed the possible benefit of SCIT in decreasing the number of asthma attacks. Another three studies reported on the number of hospitalizations with moderate heterogeneity, from which the pooled data revealed possible efficacy of SCIT in reducing the rate of hospitalizations: MD −0.07 (95% CI: −0.25, 0.12; *I*^2 ^= 56%). The sensitivity analyses were not applicable as none of the six studies were found to be at high ROB. Four articles also reported on exacerbation defined in other various ways, which we were unable to pool.

### 3.3. Bronchial provocation test (BPT)

**Figure 7:**
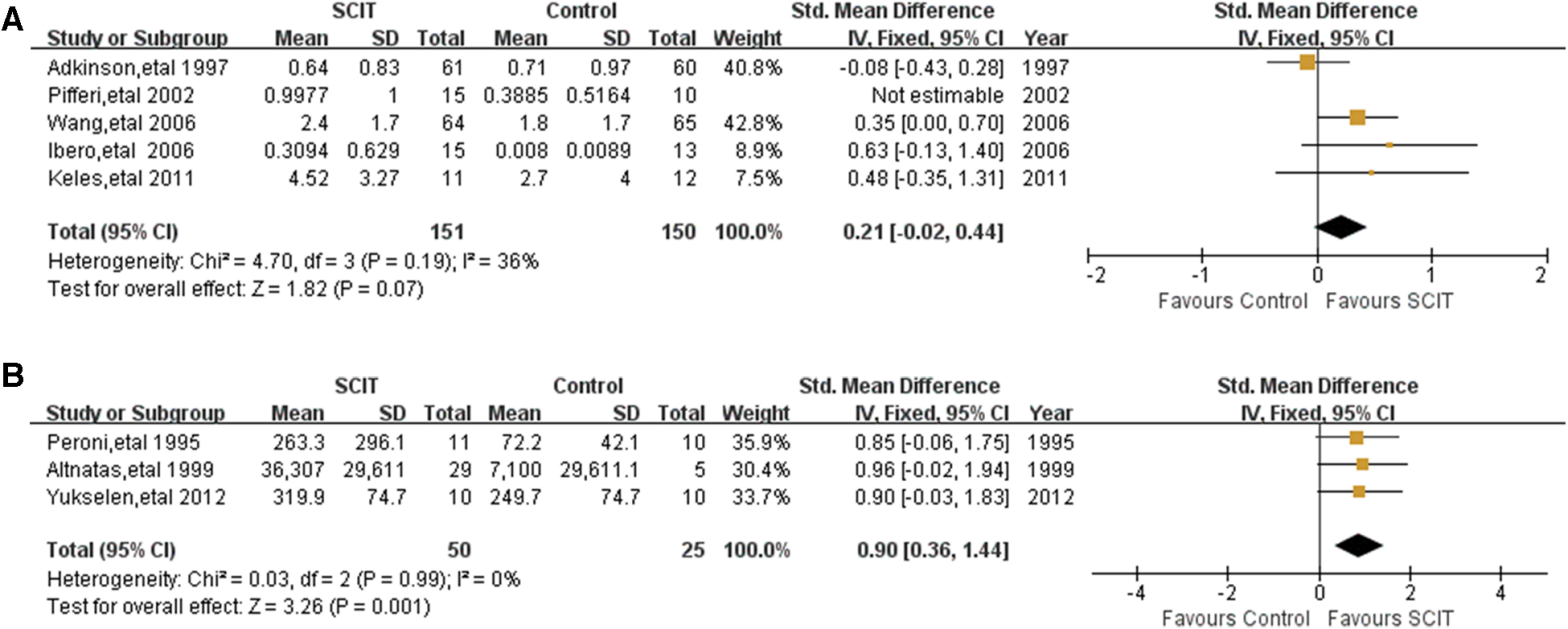
Meta-analysis of RCTs for brochial provocation test comparing SCIT and control groups: (**A**) nonspecific brochial provocation test (after sensitivity analysis; fix-effects model); (**B**) allergen-specific brochial provocation test (fix-effects model).

Five mildly heterogeneous studies reported the data of non-specific BPT defined by methacholine PC20 or histamine PC20. Pooling of those data showed an SMD of 0.25 (95% CI: 0.03, 0.46; *I*^2 ^= 33%), however turning to SMD 0.21 (95% CI: −0.02, 0.44; see Figure 7A) after sensitivity analysis, which indicates possible evidence in favor of SCIT. There were another three studies reporting the change of logPC20 or cold dry air challenge between groups without significant difference as well (16, 27, 34). Three studies performed HDM-specific BPT and the meta-analysis demonstrated an obvious benefit of SCIT with an SMD of 0.90 (95% CI: 0.36, 1.44; see Figure 7B). It appears that SCIT would have a greater effect on allergen specific airway hyperreactivity (AHR) than nonspecific AHR.

## 4. Discussion

From the pooled data we analyzed, SCIT in HDM-sensitized asthmatic children resulted in significant short-term reductions in asthma symptoms and medication use, regardless of difference in treatment duration and number of allergens sensitized. For secondary outcomes, SCIT could decrease allergen-specific AHR, but without significant improvement in pulmonary function.

Similar efficacy of SCIT in asthma with different allergens in children and adults has also been noted in previous systematic reviews or meta-analysis (6, 10, 55–57). At least three years of SCIT maintenance is recommended in some DBPC trails, which is consistent with our findings that an adequate course of treatment could provide better benefits in reducing medication use and improving PEF compared with less than three years. However, some indirect evidence in our study suggested the low efficacy of SCIT with mixed allergen extracts. The reason might be the ineffective dose concentration of the main clinically relevant allergens (55, 58). In Europe, single or few allergens (homologous only) considered to be most clinically relevant are typically used in polysensitized patients (59–61). Nevertheless, considering the prevalence of polysensitization in patients with allergic rhinoconjunctivitis (AR) or AA, furthermore, the interaction between the multiplicity of allergens and the severity of disease, there is still widespread clinical use of extract mixtures, in Unite States for example (9, 60, 62). More well-designed clinical trials comparing monoallergen or oligoallergen (2 or 3 allergens) with polyallergen immunotherapy strategies are proposed in a head-to-head approach (9). Besides, in the subgroup analysis for the moderate-severe asthma, low-grade evidence supported the ineffectiveness of SCIT in the symptom control and medication usage reduction. It might be due to the confounding factor of mixed allergens or the different phenotypic characteristics of severe asthma (63). Because of the vague population information in the original literature, we could not determine the asthma severity of all subjects and this sub-analysis of asthma severity results in population overlap. Regarding efficacy in patients with severe asthma, which is still controversial, a previous study showed that adults with moderate persistent asthma could benefit more than those with severe asthma after HDM SCIT, supporting better effect of SCIT in patients with mild to moderate asthma than the severe likewise (64). Research on patients with severe asthma but well controlled with drug treatment is still required. However, because severe asthma has been frequently reported as a risk factor for systemic adverse reactions with AIT, especially when uncontrolled, evidence of the effectiveness of AIT on severe asthma is rather limited (65–68). To reduce the risks for these patients, some new emerging therapeutic approaches have been proposed, such as the use of omalizumab (69–71). In addition, the impact of HDM SCIT on quality of life, asthma control and exacerbation in asthmatic children need to be further explored.

Because of the difficulty of performing DBPC studies in asthmatic children, the safety of SCIT was mainly validated in single-arm studies, which showed mild to moderate risk of systemic AEs [mostly Grade I-III according to WAO (72)]. Compared with the results of another systematic review involving asthmatic children and adults without restriction of SCIT allergen types (73), the risks of both local and systemic AEs were higher in our study (RR: 1.4 vs. 4.37 for local AE and RR: 2.45 vs. 2.90 for systemic AE), indicating a potential increased risk of SCIT in asthmatic children allergic to HDM. In contrast to previous studies supporting the significantly greater number of systemic reactions in children under the age of 5 (74), three studies included in this review demonstrated the favourable safety in younger children. Although this may be related to the increased experience of allergists, the result is still not unassailable due to the potentially unrepresentative sample because some studies reporting on the data of children and adults simultaneously which we had to exclude at first. Focusing on those single-arm trials with rather higher incidence of local or systemic AEs, it could be found that the majority was associated with moderate-severe asthma or rush schedule. However, given the considerable variation in methods and the lack of information in some studies, our assumption is casual and needs to be confirmed. It is imperative to offer more high-quality evidence to clarify the risk of SCIT in people of different ages and extracts with different allergen types. In the other hand, prior use of oral antihistamines may prevent the occurrence of adverse reactions. The majority of systemic reactions [86% published in a survey (75)] occurred within 30 min after injections which can be observed in the clinic and treated timely and effectively (76). Consistent with our finding, fatal anaphylaxis is rarely reported under the guidance of a professional medical team (77).

In spite of some new homogeneous conclusions that we have drawn, substantial heterogeneity remained after sensitivity analysis and subgroup analyses, especially on the primary outcomes of short-term symptom scores and medication scores. This heterogeneity can be partly explained by the different scoring schemes used in the original studies, as some objective outcomes did not show heterogeneity (e.g., MMEF or BPT) or turned to homogenous after stratifying (e.g., FEV1 for the mono-/poly-sensitization). EAACI has published the recommendation of standard criteria for the assessment of symptom scores and medication scores in AR (78). However, there is still a lack of relative documents on AA. To facilitate interpretation of future studies, standardization of asthma symptom and medication scores is urgently required. Medication requirements reported as categories may translate into a useful outcome for that (73). In addition, because of the insufficient information, we did not undertake the planned subgroup analyses of the asthma courses and the administration of allergen preparations. Based on the results of our study and previous conclusions, we assumed that a prolonged asthma course would be more likely to trend toward polysensitized state and a complex phenotype, which may preclude the benefit of AIT (62). Many studies have reported the efficacy of novel approaches of SCIT (such as rush SCIT, semi rush SCIT or cluster SCIT), however, in small sample size or open label (79, 80).

There are still some limitations to be considered in this review. Firstly, the major limitation is that we did not include all data from the potentially eligible population. Several studies involving both children and adults did not report on the relevant data separately, which we had to excluded. In addition, there were remaining 18 references we were unable to retrieve to further screen. We sought the full text or further information from the authors, but received little. Besides, literature written in languages other than English or Chinese, conference reports and potential literature in other databases were not considered in our review, which could result in publication bias. Secondly, we were unable to pool data from all retrieved articles because of the insufficient information and the heterogeneity of approaches. The results of this review, especially for secondary outcomes, may not be representative of all trials, which needs further researches to confirm.

## 5. Conclusion

SCIT can reduce asthma symptoms and medication usage and improve the allergen-specific airway hyperreactivity in asthmatic children sensitized to HDM, but with a risk of mainly mild-moderate adverse reactions. The effectiveness of SCIT on lung function, asthma control, exacerbation and long-term efficacy after discontinuation is not conclusive, both in sub-population with mixed allergens and severe asthma, which requires further investigation. Overall, SCIT is still recommended for children with mild-moderate HDM-driven allergic asthma.

## Data Availability

The original contributions presented in the study are included in the article/**Supplementary Material**, further inquiries can be directed to the corresponding author.

## Supplementary material

SUPPLEMENTARY TABLE S1

General characteristics of the Included Studies about SCIT efficacy.

SUPPLEMENTARY TABLE S2

General characteristics of the Included Studies about SCIT safety.

SUPPLEMENTARY TABLE S3

The risk of bias assessment of included randomized controlled trials.
